# Smaller spared subcortical nuclei are associated with worse post-stroke sensorimotor outcomes in 28 cohorts worldwide

**DOI:** 10.1093/braincomms/fcab254

**Published:** 2021-10-27

**Authors:** Sook-Lei Liew, Artemis Zavaliangos-Petropulu, Nicolas Schweighofer, Neda Jahanshad, Catherine E Lang, Keith R Lohse, Nerisa Banaj, Giuseppe Barisano, Lee A Baugh, Anup K Bhattacharya, Bavrina Bigjahan, Michael R Borich, Lara A Boyd, Amy Brodtmann, Cathrin M Buetefisch, Winston D Byblow, Jessica M Cassidy, Charalambos C Charalambous, Valentina Ciullo, Adriana B Conforto, Richard C Craddock, Adrienne N Dula, Natalia Egorova, Wuwei Feng, Kelene A Fercho, Chris M Gregory, Colleen A Hanlon, Kathryn S Hayward, Jess A Holguin, Brenton Hordacre, Darryl H Hwang, Steven A Kautz, Mohamed Salah Khlif, Bokkyu Kim, Hosung Kim, Amy Kuceyeski, Bethany Lo, Jingchun Liu, David Lin, Martin Lotze, Bradley J MacIntosh, John L Margetis, Feroze B Mohamed, Jan Egil Nordvik, Matthew A Petoe, Fabrizio Piras, Sharmila Raju, Ander Ramos-Murguialday, Kate P Revill, Pamela Roberts, Andrew D Robertson, Heidi M Schambra, Na Jin Seo, Mark S Shiroishi, Surjo R Soekadar, Gianfranco Spalletta, Cathy M Stinear, Anisha Suri, Wai Kwong Tang, Gregory T Thielman, Vincent N Thijs, Daniela Vecchio, Nick S Ward, Lars T Westlye, Carolee J Winstein, George F Wittenberg, Kristin A Wong, Chunshui Yu, Steven L Wolf, Steven C Cramer, Paul M Thompson, ENIGMA Stroke Recovery Working Group, Nerisa Banaj, Giuseppe Barisano, Lee Baugh, Adrià Bermudo Gallaguet, Anup Bhattacharya, Bavrina Bigjahan, Michael Borich, Lara Boyd, Amy Brodtmann, Truman Brown, Cathrin Buetefisch, Winston Byblow, Jessica Cassidy, Charalambos Charalambous, Valentina Ciullo, Alison Cloutier, James Cole, Adriana Conforto, Richard Craddock, Steven Cramer, Rosalia Dacosta Aguayo, Julie DiCarlo, Michael Dimyan, Martin Domin, Miranda Donnellly, Adrienne Dula, Matthew Edwardson, Natalia Egorova, Elsa Ermer, Mark Etherton, Wuwei Feng, Kelene Fercho, Jennifer Ferris, Fatemeh Geranmayeh, Chris Gregory, Shahram Hadidchi, Colleen Hanlon, Leticia Hayes, Kathryn Hayward, Jess Holguin, Brenton Hordacre, Darryl Hwang, Neda Jahanshad, Keith Jamison, Julia Juliano, Steven Kautz, Mohamed Salah Khlif, Bokkyu Kim, Hosung Kim, Amy Kuceyeski, Catherine Lang, Jenny Lee, Sook-Lei Liew, David Lin, Jingchun Liu, Bethany Lo, Keith Lohse, Martin Lotze, Bradley MacIntosh, John Margetis, Daniel Margulies, Maria Mataro, Keith McGregor, Feroze Mohamed, Jan Nordvik, Emily Olafson, Alexandre Perera-LLuna, Matthew Petoe, Aaron Phillips, Fabrizio Piras, Sharmila Raju, Ander Ramos-Murguialday, Kate Revill, Pamela Roberts, Andrew Robertson, Jane Rondina, Natalia Rost, Nerses Sanossian, Heidi Schambra, Christian Schranz, Nicolas Schweighofer, Na Jin Seo, Farshid Sepehrband, Mark Shiroishi, Julia Simon, Surjo Soekadar, Gianfranco Spalletta, Shraddha Srivastava, Jill Stewart, Cathy Stinear, Anisha Suri, Myriam Taga, Wai Kwong Tang, Gregory Thielman, Vincent Thijs, Sophia Thomopoulos, Paul Thompson, Daniela Vecchio, Steven Warach, Nick Ward, Emilio Werden, Lars Westlye, Roland Wiest, Carolee Winstein, George Wittenberg, Steven Wolf, Kristin Wong, Chunshui Yu, Artemis Zavaliangos-Petropulu

**Affiliations:** 1 Chan Division of Occupational Science and Occupational Therapy, University of Southern California, Los Angeles, CA, USA; 2 Keck School of Medicine, Mark and Mary Stevens Neuroimaging and Informatics Institute, University of Southern California, Los Angeles, CA, USA; 3 Neuroscience Graduate Program, University of Southern California, Los Angeles, CA, USA; 4 Imaging Genetics Center, Mark and Mary Stevens Neuroimaging and Informatics Institute, Keck School of Medicine, University of Southern California, Marina del Rey, CA, USA; 5 Biokinesiology and Physical Therapy, Ostrow School of Medicine, University of Southern California, Los Angeles, CA, USA; 6 Departments of Physical Therapy, Washington University School of Medicine, St. Louis, MO, USA; 7 Department of Occupational Therapy, Washington University School of Medicine, St. Louis, MO, USA; 8 Department of Neurology, Washington University School of Medicine, St. Louis, MO, USA; 9 Department of Health and Kinesiology, University of Utah, Salt Lake City, UT, USA; 10 Laboratory of Neuropsychiatry, IRCCS Santa Lucia Foundation, Rome, Italy; 11 Laboratory of Neuro Imaging, Mark and Mary Stevens Neuroimaging and Informatics Institute, Keck School of Medicine, University of Southern California, Los Angeles, CA, USA; 12 Basic Biomedical Sciences, Sanford School of Medicine, University of South Dakota, Vermillion, SD, USA; 13 Sioux Falls VA Health Care System, Sioux Falls, SD, USA; 14 Center for Brain and Behavior Research, Vermillion, SD, USA; 15 Sanford Research, Sioux Falls, SD, USA; 16 Mallinckrodt Institute of Radiology, Washington University School of Medicine in St. Louis, St. Louis, MO, USA; 17 Department of Radiology, Keck School of Medicine, University of Southern California, Los Angeles, CA, USA; 18 Department of Rehabilitation Medicine, Emory University, Atlanta, GA, USA; 19 Department of Physical Therapy & the Djavad Mowafaghian Centre for Brain Health, Faculty of Medicine, University of British Columbia, Vancouver, BC, Canada; 20 Florey Institute of Neuroscience and Mental Health, University of Melbourne, Heidelberg, VIC, Australia; 21 Eastern Cognitive Disorders Clinic, Monash University, Melbourne, VIC, Australia; 22 Department of Neurology, School of Medicine, Emory University, Atlanta, GA, USA; 23 Department of Radiology, Emory University, Atlanta, GA, USA; 24 Department of Exercise Sciences and Centre for Brain Research, University of Auckland, Auckland, New Zealand; 25 Allied Health Science, University of North Carolina at Chapel Hill, Chapel Hill, NC, USA; 26 Department of Basic and Clinical Sciences, University of Nicosia Medical School, Nicosia, Cyprus; 27 Center for Neuroscience and Integrative Brain Research (CENIBRE), University of Nicosia Medical School, Nicosia, Cyprus; 28 Hospital das Clínicas da Faculdade de Medicina da Universidade de São Paulo, São Paulo, SP, Brazil; 29 Hospital Israelita Albert Einstein, São Paulo, SP, Brazil; 30 Department of Neurology, Dell Medical School, University of Texas at Austin, Austin, TX, USA; 31 Melbourne School of Psychological Sciences, University of Melbourne, Melbourne, VIC, Australia; 32 Department of Health Sciences & Research, Medical University of South Carolina, Charleston, SC, USA; 33 Civil Aerospace Medical Institute, US Federal Aviation Administration, Oklahoma City, OK, USA; 34 Division of Basic Biomedical Sciences, Sanford School of Medicine, University of South Dakota, Vermillion, SD, USA; 35 Cancer Biology, Wake Forest School of Medicine, Winston Salem, NC, USA; 36 College of Health Professions, Medical University of South Carolina, Charleston, SC, USA; 37 Department of Physiotherapy, University of Melbourne, Heidelberg, VIC, Australia; 38 NHMRC CRE in Stroke Rehabilitation and Brain Recovery, University of Melbourne, Heidelberg, VIC, Australia; 39 Innovation, IMPlementation and Clinical Translation (IIMPACT) in Health, Allied Health and Human Performance, University of South Australia, Adelaide, SA, Australia; 40 Department of Biomedical Engineering, Viterbi School of Engineering, University of Southern California, Los Angeles, CA, USA; 41 Ralph H. Johnson VA Medical Center, Charleston, SC, USA; 42 Department of Physical Therapy Education, College of Health Professions, SUNY Upstate Medical University, Syracuse, NY, USA; 43 Department of Radiology, Weill Cornell Medicine, New York, NY, USA; 44 Department of Radiology, Tianjin Medical University General Hospital, Tianjin, China; 45 Center for Neurotechnology and Neurorecovery, Department of Neurology, Massachusetts General Hospital, Boston, MA, USA; 46 Department of Diagnostic Radiology, University Medicine Greifswald, Greifswald, Germany; 47 Hurvitz Brain Sciences, Sunnybrook Research Institute, Toronto, ON, Canada; 48 Department of Medical Biophysics, University of Toronto, Toronto, ON, Canada; 49 Jefferson Integrated Magnetic Resonance Imaging Center, Department of Radiology, Thomas Jefferson University, Philadelphia, PA, USA; 50 CatoSenteret Rehabilitation Center, Son, Norway; 51 Bionics Institute, Melbourne, VIC, Australia; 52 Department of Medicine and Centre for Brain Research, University of Auckland, Auckland, New Zealand; 53 Department of Neurology, New York University Langone, New York, NY, USA; 54 TECNALIA, Basque Research and Technology Alliance (BRTA), Health Division, San Sebastian Donostia, Spain; 55 Institute of Medical Psychology and Behavioral Neurobiology, University of Tübingen, Tübingen, Germany; 56 Facility for Education and Research in Neuroscience, Emory University, Atlanta, GA, USA; 57 Department of Physical Medicine and Rehabilitation, Cedars-Sinai, Los Angeles, CA, USA; 58 California Rehabilitation Institute, Los Angeles, CA, USA; 59 Canadian Partnership for Stroke Recovery, Sunnybrook Research Institute, University of Toronto, Toronto, ON, Canada; 60 Department of Kinesiology, University of Waterloo, Waterloo, ON, Canada; 61 Department of Rehabilitation Sciences, Medical University of South Carolina, Charleston, SC, USA; 62 Clinical Neurotechnology Laboratory, Department of Psychiatry and Psychotherapy, Charité - University Medicine Berlin, Berlin, Germany; 63 Division of Neuropsychiatry, Menninger Department of Psychiatry and Behavioral Sciences, Baylor College of Medicine, Houston, TX, USA; 64 Department of Medicine, University of Auckland, Auckland, New Zealand; 65 Department of Electrical and Computer Engineering, Swanson School of Engineering, University of Pittsburgh, Pittsburgh, PA, USA; 66 Department of Psychiatry, Faculty of Medicine, the Chinese University of Hong Kong, Hong Kong, China; 67 Department of Physical Therapy and Neuroscience, University of the Sciences, Philadelphia, PA, USA; 68 Department of Neurology, Austin Health, Heidelberg, VIC, Australia; 69 UCL Queen Square Institute of Neurology, London, UK; 70 Department of Psychology, University of Oslo, Oslo, Norway; 71 NORMENT, Division of Mental Health and Addiction, Oslo University Hospital, Oslo, Norway; 72 Department of Neurology, University of California, Los Angeles, Los Angeles, CA, USA; 73 Department of Neurology, University of Pittsburgh, Pittsburgh, PA, USA; 74 Neurology, Department of Veterans Affairs Pittsburgh Healthcare System, Pittsburgh, PA, USA; 75 Department of Physical Medicine & Rehabilitation, Dell Medical School, University of Texas at Austin, Austin, TX, USA; 76 Tianjin Key Laboratory of Functional Imaging, Tianjin Medical University General Hospital, Tianjin, China; 77 Division of Physical Therapy Education, Department of Rehabilitation Medicine, Emory University School of Medicine, Atlanta, GA, USA; 78 Division of Physical Therapy Education, Department of Medicine, Emory University School of Medicine, Atlanta, GA, USA; 79 Division of Physical Therapy Education, Department of Cell Biology, Emory University School of Medicine, Atlanta, GA, USA; 80 Nell Hodgson Woodruff School of Nursing, Emory University, Atlanta, GA, USA; 81 Center for Visual and Neurocognitive Rehabilitation, Atlanta VA Health Care System, Decatur, GA, USA

**Keywords:** stroke, rehabilitation, sensorimotor behaviour, MRI, subcortical volumes

## Abstract

Up to two-thirds of stroke survivors experience persistent sensorimotor impairments. Recovery relies on the integrity of spared brain areas to compensate for damaged tissue. Deep grey matter structures play a critical role in the control and regulation of sensorimotor circuits. The goal of this work is to identify associations between volumes of spared subcortical nuclei and sensorimotor behaviour at different timepoints after stroke. We pooled high-resolution T_1_-weighted MRI brain scans and behavioural data in 828 individuals with unilateral stroke from 28 cohorts worldwide. Cross-sectional analyses using linear mixed-effects models related post-stroke sensorimotor behaviour to non-lesioned subcortical volumes (Bonferroni-corrected, *P* < 0.004). We tested subacute (≤90 days) and chronic (≥180 days) stroke subgroups separately, with exploratory analyses in early stroke (≤21 days) and across all time. Sub-analyses in chronic stroke were also performed based on class of sensorimotor deficits (impairment, activity limitations) and side of lesioned hemisphere. Worse sensorimotor behaviour was associated with a smaller ipsilesional thalamic volume in both early (*n* = 179; *d *=* *0.68) and subacute (*n* = 274, *d *=* *0.46) stroke. In chronic stroke (*n* = 404), worse sensorimotor behaviour was associated with smaller ipsilesional putamen (*d *=* *0.52) and nucleus accumbens (*d *=* *0.39) volumes, and a larger ipsilesional lateral ventricle (*d *=* *−0.42). Worse chronic sensorimotor impairment specifically (measured by the Fugl-Meyer Assessment; *n* = 256) was associated with smaller ipsilesional putamen (*d *=* *0.72) and larger lateral ventricle (*d* = −0.41) volumes, while several measures of activity limitations (*n* = 116) showed no significant relationships. In the full cohort across all time (*n* = 828), sensorimotor behaviour was associated with the volumes of the ipsilesional nucleus accumbens (*d *=* *0.23), putamen (*d *=* *0.33), thalamus (*d *=* *0.33) and lateral ventricle (*d* = −0.23). We demonstrate significant relationships between post-stroke sensorimotor behaviour and reduced volumes of deep grey matter structures that were spared by stroke, which differ by time and class of sensorimotor measure. These findings provide additional insight into how different cortico-thalamo-striatal circuits support post-stroke sensorimotor outcomes.

## Teaser

Liew et al. report the first large-scale examination using high-resolution neuroimaging of subcortical nuclei and sensorimotor behaviour in 828 stroke patients from 28 cohorts worldwide. They discovered novel associations between post-stroke sensorimotor behaviour and specific subcortical nuclei, providing new insight for stroke rehabilitation.

## Graphical Abstract

**Graphical Abstract:**
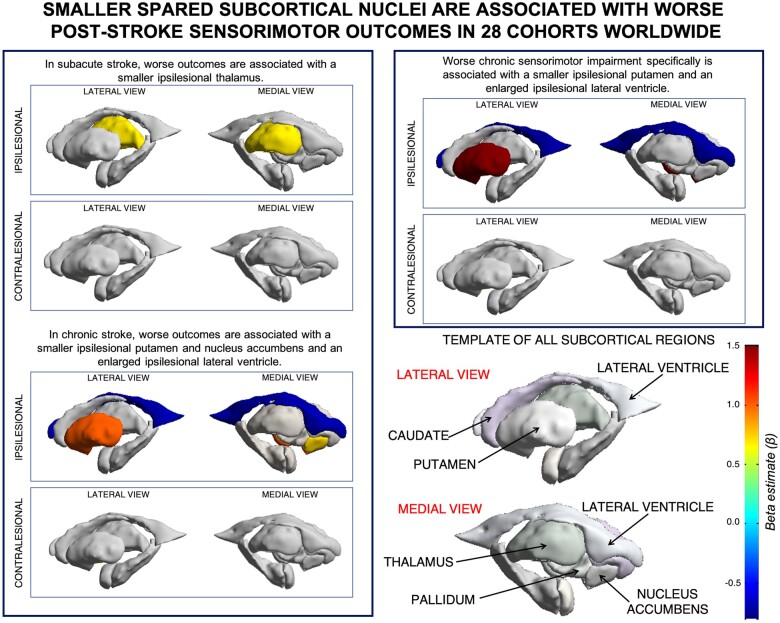

## Introduction

Sensorimotor recovery after stroke relies on residual motor architecture.^1^ The majority of research in this area has focused on the role of cortical regions within sensorimotor networks, which often undergo significant reorganization and vary widely across individuals after stroke. Spared subcortical nuclei also form key components of corticothalamic and corticostriatal circuits that support sensorimotor performance but have been less studied in recent years. These structures may yield additional insight into processes impacting stroke outcomes, given their clearly defined boundaries, well-mapped inputs and outputs, and known associations with specific neurotransmitters and genetic variants.^2^

As relay nodes for sensorimotor circuits in the brain, subcortical nuclei not only play a critical role in the maintenance and regulation of networks for motor learning, but they also subserve cognition, metabolic regulation, and reward—all of which have been implicated as contributors to post-stroke outcomes, including sensorimotor functioning and recovery.^3–6^ Each structure in the cortico-striatal-thalamic circuit has a distinct role in sensorimotor control and possibly outcomes. For instance, the thalamus is integral to the regulation of metabolism, sleep and wakefulness, cognitive processing, and integrating sensorimotor information,^7^ and thalamic metabolism has been shown to be disordered in the early weeks after stroke.^3^^,^^8^ Similarly, the basal ganglia (e.g. caudate, putamen, globus pallidus, and nucleus accumbens) are heavily involved in motor control, learning and reward, with distinct roles for each nuclei.^9^^,^^10^ Direct damage to the thalamus and basal ganglia is associated with poor sensorimotor behaviour and recovery,^4^^,^^11^ but the role of each spared subcortical nuclei is unclear.

To date, these subcortical structures have been studied only in modestly-sized samples, with varying results, and with measurements from multiple regions often aggregated as one (e.g. combined analysis of the thalamus and basal ganglia). However, each nucleus has a characteristic distribution of neurotransmitters and network connections; identifying specific non-lesioned subcortical nuclei could provide more precise neurobiological targets for therapeutics to potentiate recovery.

In addition, inter-individual variability and the heterogeneity of brain changes after stroke pose challenges to the identification of neural targets in spared tissue. Addressing this issue requires large, diverse, and appropriately powered sample sizes with high-resolution brain MRIs. Although acute stroke research has successfully utilized pooled approaches with individual patient data to examine acute treatment outcomes,^12^^,^^13^ stroke rehabilitation research has been slower to adopt this type of approach due to the complexity of combining elaborate rehabilitation research protocols, differences in the site and size of infarcts, diversity of the patient populations recruited, and variety of the stroke neuroimaging and behavioural measures collected. To address these challenges, we formed an international Stroke Recovery Working Group through the Enhancing NeuroImaging Genetics through Meta-Analysis (ENIGMA) Consortium to harmonize and combine diverse individual patient data, including high-resolution structural brain MRIs and behavioural outcome measures, across multiple research centres.^14^ Our ENIGMA Stroke Recovery Working Group pools individual patient data across research sites using a harmonized analytical pipeline and includes both published and unpublished data. Compared to traditional single-site analyses or retrospective meta-analyses, this approach allows for greater statistical rigour, testing of more sophisticated hypotheses (e.g. subgroup analyses), and less bias due to the inclusion of both published and unpublished data across diverse cohorts.^15^^,^^16^ Furthermore, pooled analyses with multi-site data increase heterogeneity, which improves generalizability of findings, reduces research inefficiency by leveraging previously collected data to examine novel questions, and advances the field faster than is achievable by prospective studies.^17^

The current study pools data from 828 individuals across 28 cohorts worldwide from the ENIGMA Stroke Recovery Working Group to examine relationships between sensorimotor behavioural measures and volumes of the ipsilesional and contralesional thalamus, putamen, caudate, pallidum and nucleus accumbens. Enlargement of the lateral ventricles was also examined as an indirect measure of atrophy and vascular integrity.^18^^,^^19^ Given the neurobiological events unique to early and subacute stroke compared to chronic stroke, data were analysed separately for individuals in the subacute (≤90 days) and chronic (≥180 days) stages.^20^ As an exploratory measure, we also analysed relationships early after stroke (≤21 days), before post-stroke secondary structural atrophy is thought to be observed,^21^ to estimate whether subacute associations are driven by early post-stroke changes or likely existed prior to the stroke, as well as across all time.

We hypothesized that thalamic volume would relate to sensorimotor behaviour in early and subacute phases after stroke, given its multiple roles in supporting cellular repair.^3^^,^^22^ We further expected that smaller subcortical volumes (reflecting atrophy of structures associated with sensorimotor control) and larger ventricles (reflecting general atrophy) would be related to worse chronic sensorimotor behaviour.^23^

Furthermore, as sensorimotor behaviour encompasses multiple classes of the International Classification of Functioning, Disability, and Health (ICF), we conducted separate subgroup analyses in chronic stroke to examine if there are specific neural correlates of loss of body structures and function (i.e. *sensorimotor impairment*) versus loss of activity in daily tasks (i.e. *activity limitations*).^24^ We anticipated that subcortical nuclei important for direct sensorimotor control, such as the putamen, would more strongly relate to impairment; conversely, regions associated with reward and motivation, such as the nucleus accumbens, should more strongly relate to activity limitation. Finally, in chronic stroke, we also examined the impact of the side of the lesion. Based on evidence of hemispheric specialization for motor behaviour after stroke,^25^ we hypothesized that the side of the lesion would modify the relationship between non-lesioned subcortical tissue volume and sensorimotor behaviour.

## Materials and methods

### Study design

The current cross-sectional pooled analysis used data from the ENIGMA Stroke Recovery Working Group, which was frozen for this analysis on 22 May 2020. A detailed overview of ENIGMA Stroke Recovery procedures and methods are reported elsewhere.^14^ The retrospective data were collected across 28 different research studies (i.e. cohorts) at 16 different research institutes in 10 countries. Data were collected in accordance with the Declaration of Helsinki and in compliance with local ethics review boards at each institute (see Supplementary Table 1 for details).

### ENIGMA stroke recovery dataset

Participants with at least one sensorimotor behavioural outcome measure (see Behavioural Data Analysis) and a segmented high-resolution (e.g. 1-mm isotropic) T_1_-weighted (T1w) structural MRI of the brain (see MRI data analysis) were included, yielding an initial dataset of 1285 individuals. Only participants with unilateral ischaemic stroke or intracerebral haemorrhage in subcortical and/or cortical regions were included, while individuals identified as having bilateral lesions or lesions in the brainstem or cerebellum were excluded from this analysis. For any longitudinal observations, only the first time-point was used; the resulting dataset was therefore cross-sectional. Each brain region was manually inspected for quality and overlap with the lesion (see MRI data analysis)*.* Any individuals missing covariates of age (*n* = 50) or sex (*n* = 89) were also excluded, yielding a final sample of 828 individuals. As the relationships between brain volume and sensorimotor behaviour were expected to change with time after stroke, the data were divided into subacute stroke (≤90 days post-stroke) and chronic stroke (≥180 days post-stroke). Exploratory analyses looking only at early stroke (≤21 days post-stroke) and across all times after stroke are also included.

### MRI data analysis

To extract subcortical volumes, the brain imaging software package FreeSurfer (version 5.3) was used to segment subcortical regions of interest (ROIs) from the T1w MRIs.^26^ Twelve ROIs were extracted: the left and right thalamus, caudate, putamen, pallidum, nucleus accumbens and lateral ventricles. For all analyses, these were characterized as ipsilesional and contralesional with respect to the lesioned hemisphere. Total intracranial volume (ICV) was also quantified using FreeSurfer outputs. ENIGMA scripts developed in-house were used to extract the volume of each ROI for each individual and to generate quality control triplanar images of each segmented ROI as done previously (http://enigma.ini.usc.edu/protocols/).^2^ Given the variability of post-stroke neuroanatomy following a lesion, trained research team members (A.Z.-P., A.S.) performed visual quality control for each ROI in each subject. Any regions intersecting the lesion were marked ‘lesioned’, and any regions not properly segmented by FreeSurfer were marked ‘failed’. Regions falling in either category were excluded from further analysis (for the full quality control protocol, see Appendix 1 in ref 14). Sample sizes for each analysis and brain region are reported in each results table.

### Behavioural data analysis

Across cohorts, behavioural data were collected within approximately 72 h of the MRI. To maximize the utility of the full dataset, a *primary sensorimotorbehaviourscore* was defined for each study cohort using the standardized measure reported by that cohort which was most commonly represented in the dataset overall (see Supplementary materials). In order to aggregate the different measures across cohorts, for each measure, a fraction of the maximum possible score was calculated, such that 0 represented the worst sensorimotor performance (severe deficits) and 1 represented the best sensorimotor performance (no deficits). For example, the most common measure used across cohorts was the Fugl-Meyer Motor Assessment of Upper Extremities (FMA-UE),^27^ and a score of 45 out of the maximum 66 possible points on this assessment would be represented as 0.68.

In chronic stroke, we also examined behavioural measures that specifically captured impairment and activity limitation. Impairment was measured by the FMA-UE, whereas activity limitation was measured by the Action Research Arm Test (ARAT)^28^ and Wolf Motor Function Test (WMFT).^29^ These data were not examined in early stroke due to the limited sample sizes with these measures.

### Statistical analysis

To examine the relationships between sensorimotor behaviour and non-lesioned subcortical volumes, we performed linear mixed-effects regressions. A separate regression model was run for the volume of each subcortical ROI (outcome) using sensorimotor behaviour (e.g. primary sensorimotor behaviour score, sensorimotor impairment, or activity limitations) as the primary predictor of interest. After ruling out collinearity (variance inflation factor ≤ 2.5), normalized age, ICV, and sex were included as fixed effects. Research cohort was included as a random effect. In chronic stroke, the effect of lesioned hemisphere was examined by including an interaction term between sensorimotor behaviour and side of lesioned hemisphere to the model predicting subcortical volume. This was not examined in subacute stroke due to the smaller sample size. A likelihood ratio test was performed to compare models with and without random effects and showed that the random effects were always significant. The regression assumptions of linearity, normality of the residuals, and homogeneity of the residual variance were checked via visual inspection of residuals versus fits plots as well as qq-plots for both individual observations and research cohorts. Potential influential values for both observations and cohorts were assessed using Cook’s distance with recommended thresholds.^30^ As we detected influential observations in almost all analyses, we re-ran the analyses using robust mixed-effect regression, which reduces the weight of influential observations in the models without excluding data.^31^ Results did not differ between original and robust regression models. The results of the robust regression models can be found in Supplementary materials.

For all regression analyses, beta coefficients are presented for the predictor of interest (e.g. sensorimotor behaviour, sensorimotor impairment, or activity limitations), along with the sample size (n), standard error (SE), 95% confidence interval (CI), degrees of freedom (df), standardized effect size (*d*), *t*-value and uncorrected *P*-value. Statistical significance was adjusted for multiple comparisons across the 12 ROIs using a Bonferroni correction (*P* < 0.004). Any significant fixed covariates are also reported.

We also compared sensorimotor behaviour scores between left and right hemisphere stroke groups. The data violated the Wilk–Shapiro test of normality for both groups (LHS: *W* = 0.89, *P* < 0.001, RHS: *W* = 0.89, *P* < 0.001). We therefore used a nonparametric Wilcoxon rank sum test to compare independent group samples.

All statistical analyses were conducted in R (version 3.6.3; R Core Team, 2020).^32^ The follow R libraries were used for the statistical analyses: the *lme* function from *nmle* was used for the linear mixed-effects regressions,^33^ the *rlmer* function from *robustlmm* was used for the robust linear mixed-effects regressions,^34^ and the *rstatix* library was used for the Wilcoxon rank sum test.^35^ In addition, *influence*. *ME* was used to detect influential values^36^ and *dplyr*^37^ and *tidyverse*^38^ libraries were used for data organization.

### Data availability

The deidentified summary data and code that support the findings of this study are available upon reasonable request from the corresponding author. The data are not all publicly available in a repository as they may contain information that could compromise the privacy of research participants. There are also data sharing restrictions imposed by some of the (i) ethical review boards of the participating sites, and consent documents; (ii) national and trans-national data sharing laws; and (iii) institutional processes, some of which require a signed data transfer agreement for limited and predefined data use. However, we welcome sharing data with researchers, requiring that they become members of the ENIGMA Stroke Recovery working group and submit an analysis plan for a secondary project for group review. Once this analysis plan is approved, access to the relevant data will be provided contingent on data availability, local PI approval and compliance with all supervening regulatory boards.

## Results

Data from 828 individuals across 28 cohorts worldwide were included (see Table  1 for an overview of cohort characteristics). Briefly, the median age was 63 years old (interquartile range (IQR) 19 years), and there were 516 males and 312 females.

**Table 1:** Summary of research cohort characteristics

Cohort ID	*n*	Females/Males	Median age (IQR, min–max)	Median sensorimotor score (IQR, min–max)
1	39	10/29	61 (17, 31–80)	0.65 (0.23, 0.0–0.9)
2	12	06/06	70 (12, 39–85)	0.50 (0.41, 0.2–0.7)
3	14	06/08	60 (15, 33–85)	0.25 (0.22, 0.1–0.6)
4	19	06/13	44 (15, 30–68)	0.14 (0.17, 0.0–0.5)
7	42	14/28	56 (14, 18–80)	0.82 (0.35, 0.4–1.0)
8	8	02/06	62 (10, 39–75)	0.55 (0.35, 0.0–1.0)
9	93	29/64	70 (16, 24–88)	1.00 (0.07, 0.0–1.0)
10	24	05/19	59 (13, 42–74)	1.00 (0.02, 0.7–1.0)
11	29	10/19	57 (11, 44–71)	1.00 (0.05, 0.1–1.0)
12	57	31/26	71 (17, 31–97)	0.65 (0.71, 0.0–1.0)
13	44	22/22	72 (18, 33–91)	0.12 (0.32, 0.0–1.0)
15	14	06/08	57 (11, 45–74)	0.72 (0.25, 0.4–0.8)
17	16	05/11	59 (04, 45–68)	0.55 (0.23, 0.2–0.7)
18	11	05/06	59 (07, 46–73)	0.65 (0.22, 0.5–0.9)
19	13	03/10	62 (21, 33–74)	0.84 (0.08, 0.8–0.9)
20	22	08/14	70 (13, 49–79)	0.91 (0.14, 0.3–1.0)
22	17	04/13	59 (30, 25–72)	0.63 (0.50, 0.0–0.8)
23	13	07/06	58 (08, 31–90)	0.42 (0.17, 0.3–0.8)
24	21	11/10	63 (13, 32–78)	0.95 (0.00, 0.6–1.0)
25	26	10/16	65 (18, 37–88)	0.97 (0.20, 0.0–1.0)
26	24	14/10	49 (20, 25–71)	0.64 (0.14, 0.3–0.8)
28	26	07/19	62 (11, 23–75)	0.75 (0.25, 0.3–1.0)
31	35	09/26	58 (12, 21–86)	0.52 (0.31, 0.2–0.9)
32	7	03/04	62 (16, 38–72)	0.95 (0.44, 0.2–1.0)
34	15	06/09	58 (11, 32–80)	0.82 (0.20, 0.6–1.0)
35	15	06/09	64 (18, 31–83)	0.64 (0.52, 0.2–0.9)
38	81	34/47	66 (19, 30–89)	0.85 (0.60, 0.0–1.0)
41	91	33/58	70 (15, 32–89)	1.00 (0.02, 0.8–1.0)
Total	828	312/516	63 (19, 18–97)	0.82 (0.48, 0–1)

Age and sensorimotor behavioural score data are shown as median [interquartile range (IQR), minimum–maximum values].

**Figure 1:**
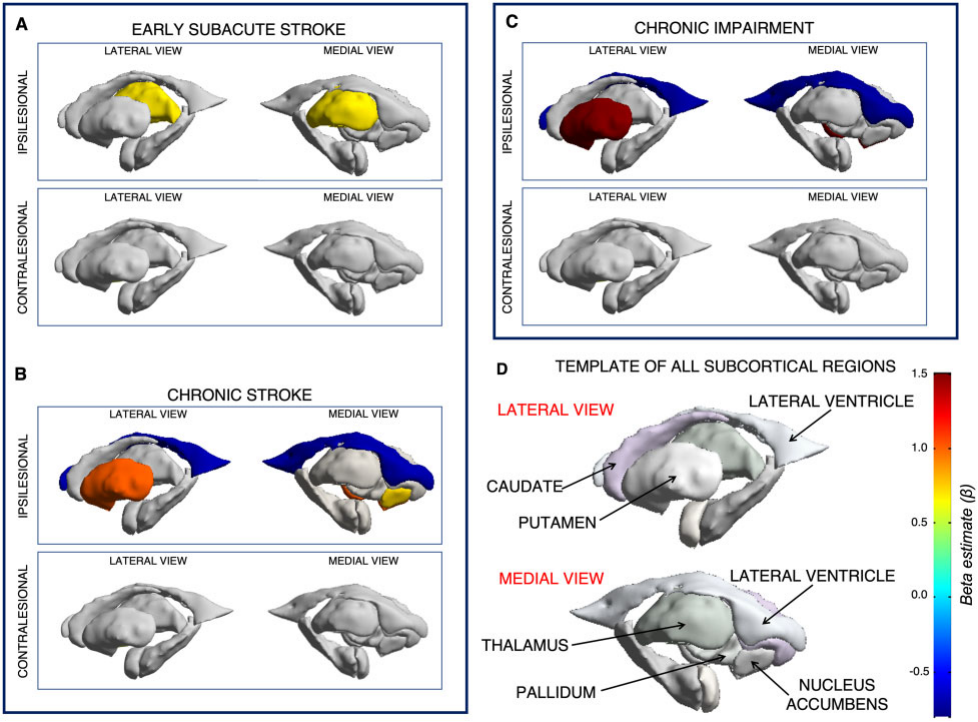
**Relationships between post-stroke sensorimotor behaviour and non-lesioned subcortical volumes.** Non-lesioned subcortical regions (**D**, bottom right) that relate to sensorimotor behaviour from linear mixed-effects models of people with subacute (**A**, top left) and chronic (**B**, bottom left) stroke. Non-lesioned subcortical volume relationships with chronic sensorimotor impairment are shown in **C** (top right). There were no significant volume relationships with chronic activity limitations. Colours represent the beta estimate (β) for sensorimotor behaviour from each model. Warmer colours represent stronger positive relationships (e.g. larger brain volumes relate to better behaviour), and cooler colours represent stronger negative relationships (e.g. larger brain volumes relate to worse behaviour).

In subacute stroke (≤90 days; *n* = 274), worse post-stroke sens orimotor behaviour was significantly associated with smaller volumes of the ipsilesional thalamus (*n* = 274, *d *=* *0.46, *P* = 0.002; Table  2; Fig.  1). Analysis of only individuals within just the first 21 days post-stroke (*n* = 179, *d *=* *0.68, *P* < 0.001) demonstrated the same result with a stronger effect (Table  2).

**Table 2:** Relationships between non-lesioned subcortical volumes and sensorimotor behaviour in subacute and early stroke

Subacute and early stroke								
Brain region	*n*	Beta (CI)	SE	df	*t*-value	*P*-value	*d*	Significant covariates
Subacute stroke (≤90 days)								
Ipsilesional								
Caudate	194	−0.01 (−0.51 to 0.48)	0.25	180	−0.06	0.954	−0.01	ICV
Lateral ventricle	274	0.18 (−0.14 to 0.51)	0.16	259	1.13	0.258	0.14	Age, ICV
Nucleus accumbens	245	0.24 (−0.14 to 0.62)	0.19	231	1.26	0.210	0.17	Age
Pallidum	223	0.21 (−0.26 to 0.67)	0.24	209	0.87	0.387	0.12	ICV
Putamen	201	0.39 (−0.09 to 0.88)	0.25	187	1.61	0.109	0.24	Age, ICV
**Thalamus**	**210**	**0.69 (0.27–1.11)**	**0.21**	**197**	**3.21**	**0.002**	**0.46**	**Age, ICV**
Contralesional								
Caudate	219	0.22 (−0.20 to 0.64)	0.21	205	1.04	0.298	0.15	ICV
Lateral ventricle	274	0.15 (−0.18 to 0.49)	0.17	259	0.92	0.361	0.11	Age, ICV
Nucleus accumbens	253	0.15 (−0.23 to 0.52)	0.19	239	0.77	0.443	0.10	Age, ICV
Pallidum	250	0.50 (0.07–0.92)	0.22	236	2.30	0.022	0.30	ICV
Putamen	229	0.37 (−0.05 to 0.79)	0.21	215	1.75	0.081	0.24	Age, ICV
Thalamus	217	0.09 (−0.33 to 0.50)	0.21	204	0.41	0.679	0.06	Age, ICV
Early stroke (≤21 days)								
Ipsilesional								
Caudate	135	−0.09 (−0.67 to 0.48)	0.29	125	−0.32	0.749	−0.06	ICV
Lateral ventricle	182	0.25 (−0.11 to 0.61)	0.18	172	1.37	0.173	0.21	Age, ICV
Nucleus accumbens	165	0.19 (−0.23 to 0.60)	0.21	155	0.90	0.369	0.14	Age
Pallidum	157	0.12 (−0.39 to 0.63)	0.26	147	0.46	0.644	0.08	ICV
Putamen	143	0.25 (−0.28 to 0.79)	0.27	133	0.93	0.354	0.16	Age, ICV
**Thalamus**	**137**	**0.79 (0.38–1.20)**	**0.21**	**128**	**3.82**	**<0.001**	**0.68**	**Age, ICV**
Contralesional								
Caudate	147	0.17 (−0.29 to 0.64)	0.24	137	0.74	0.461	0.13	ICV
Lateral ventricle	182	0.19 (−0.20 to 0.57)	0.19	172	0.96	0.337	0.15	Age, ICV
Nucleus accumbens	170	0.30 (−0.09 to 0.69)	0.20	160	1.53	0.127	0.24	Age
Pallidum	171	0.65 (0.19–1.11)	0.23	161	2.79	0.006	0.44	ICV
Putamen	158	0.26 (−0.21 to 0.72)	0.24	148	1.10	0.274	0.18	Age, ICV
Thalamus	150	0.20 (−0.28 to 0.67)	0.24	141	0.82	0.411	0.14	Age, ICV

Results from linear mixed-effects models of individuals with subacute stroke (top) and early stroke (bottom). Results in bold indicate significance with a Bonferroni correction for multiple comparisons (*P* < 0.004). The beta coefficient for sensorimotor behaviour (beta) with 95% confidence interval (CI), along with the sample size (*n*), standard error (SE), degrees of freedom (df), standardized effect size (*d*), *t*-value and uncorrected *P*-value are reported, in addition to significant fixed covariates, including age, sex and intracranial volume (ICV).

In chronic stroke (≥180 days; *n* = 404), worse sensorimotor behaviour was related to smaller volumes of the ipsilesional putamen (*d *=* *0.52, *P* < 0.001) and ipsilesional nucleus accumbens (*d *=* *0.39, *P* = 0.002), and a larger volume of the ipsilesional lateral ventricle (*d* = −0.42, p < 0.001; Table  3; Fig.  1).

**Table 3:** Relationships between non-lesioned subcortical volumes and sensorimotor behaviour in chronic stroke

Chronic stroke								
Chronic stroke (≥180 days)								
Brain region	*n*	Beta (CI)	SE	df	*t*-value	*P*-value	*d*	Significant covariates
Ipsilesional								
Caudate	193	0.27 (−0.28 to 0.82)	0.28	169	0.98	0.330	0.15	ICV
**Lateral ventricle**	**404**	**−0.70 (−1.04 to 0.36)**	**0.17**	**378**	**−4.04**	**<0.001**	**−0.42**	**Age, ICV**
**Nucleus accumbens**	**289**	**0.72 (0.27–1.18)**	**0.23**	**264**	**3.15**	**0.002**	**0.39**	**Age**
Pallidum	225	0.30 (−0.23 to 0.84)	0.27	200	1.11	0.267	0.16	ICV
**Putamen**	**207**	**1.01 (0.45–1.57)**	**0.28**	**183**	**3.54**	**<0.001**	**0.52**	**Age**
Thalamus	169	0.08 (−0.60 to 0.75)	0.34	146	0.22	0.827	0.04	Age
Contralesional								
Caudate	345	0.08 (−0.31 to 0.48)	0.20	320	0.41	0.679	0.05	ICV
Lateral ventricle	404	−0.39 (−0.70 to 0.07)	0.16	378	−2.42	0.016	−0.25	Age, ICV
Nucleus accumbens	344	0.21 (−0.22 to 0.65)	0.22	319	0.96	0.339	0.11	Age
Pallidum	359	0.20 (−0.20 to 0.60)	0.20	334	0.97	0.332	0.11	Sex, ICV
Putamen	355	0.21 (−0.18 to 0.60)	0.20	330	1.06	0.291	0.12	Age, ICV
Thalamus	329	−0.24 (−0.60 to 0.12)	0.18	304	−1.29	0.196	−0.15	Age, ICV

Results from linear mixed-effects models of individuals with chronic stroke. Results in bold indicate significance with a Bonferroni correction for multiple comparisons (*P* < 0.004). The beta coefficient for sensorimotor behaviour (beta) with 95% confidence interval (CI), along with the sample size (*n*), standard error (SE), degrees of freedom (df), standardized effect size (*d*), *t*-value and uncorrected *P*-value are reported, in addition to significant fixed covariates, including age, sex and intracranial volume (ICV).

In chronic stroke, we examined brain–behaviour relationships using a measure of impairment (the FMA-UE scale; *n* = 256) and two measures of activity limitation (WMFT, ARAT; *n* = 116). Worse sensorimotor impairment was associated with smaller ipsilesional putamen (*d *=* *0.72, *P* = 0.001) and larger ipsilesional lateral ventricle volumes (*d* = −0.41, *P* = 0.002; Table  4; Fig.  1). We found no significant relationships between subcortical volumes and measures of activity limitations (Table  4).

**Table 4:** Relationships between non-lesioned subcortical volumes and two measures of sensorimotor behaviour (impairment, activity limitations)

Chronic sensorimotor impairment and activity limitations								
Brain region	*n*	Beta (CI)	SE	df	*t*-value	*P*-value	*d*	Significant covariates
Sensorimotor impairment in chronic stroke								
Ipsilesional								
Caudate	94	0.92 (−0.06 to 1.89)	0.49	77	1.87	0.065	0.43	ICV
**Lateral ventricle**	**256**	**−0.74 (−1.20 to 0.27)**	**0.24**	**237**	−**3.13**	**0.002**	−**0.41**	**Age, ICV**
Nucleus accumbens	171	0.58 (0.01–1.15)	0.29	153	2.02	0.045	0.33	Age
Pallidum	120	0.76 (0.01–1.51)	0.38	102	2.02	0.046	0.40	–
**Putamen**	**104**	**1.50 (0.61–2.39)**	**0.45**	**87**	**3.34**	**0.001**	**0.72**	**–**
Thalamus	84	0.33 (−0.72 to 1.38)	0.53	68	0.62	0.537	0.15	–
Contralesional								
Caudate	222	0.06 (−0.44 to 0.57)	0.26	204	0.25	0.806	0.03	ICV
Lateral ventricle	256	−0.51 (−0.88 to 0.14)	0.19	237	−2.70	0.007	−0.35	Age, ICV
Nucleus accumbens	222	0.21 (−0.31 to 0.73)	0.26	204	0.80	0.425	0.11	Age
Pallidum	231	0.20 (−0.33 to 0.73)	0.27	213	0.74	0.459	0.10	Sex
Putamen	229	0.10 (−0.38 to 0.58)	0.24	211	0.41	0.681	0.06	Age, ICV
Thalamus	211	−0.40 (−0.88 to 0.07)	0.24	193	−1.67	0.096	−0.24	Age, ICV
Activity limitations in chronic stroke								
Ipsilesional								
Caudate	52	−0.63 (−1.80 to 0.53)	0.58	44	−1.09	0.280	−0.33	–
Lateral ventricle	116	−0.71 (−1.46 to 0.04)	0.38	108	−1.88	0.062	−0.36	Age, ICV
Nucleus accumbens	86	0.77 (−0.31 to 1.85)	0.54	78	1.42	0.159	0.32	–
Pallidum	64	0.71 (−0.25 to 1.67)	0.48	56	1.47	0.146	0.39	–
Putamen	65	0.71 (−0.62 to 2.04)	0.67	57	1.06	0.292	0.28	–
Thalamus	56	0.94 (−0.36 to 2.25)	0.65	48	1.45	0.153	0.42	–
Contralesional								
Caudate	96	−0.07 (−0.98 to 0.84)	0.46	88	−0.15	0.885	−0.03	–
Lateral ventricle	116	−0.72 (−1.44 to 0.01)	0.37	108	−1.95	0.054	−0.38	Age, ICV
Nucleus accumbens	107	−0.34 (−1.17 to 0.49)	0.42	99	−0.81	0.420	−0.16	Age
Pallidum	103	−0.15 (−0.98 to 0.68)	0.42	95	−0.35	0.728	−0.07	Sex
Putamen	100	0.06 (−0.91 to 1.03)	0.49	92	0.12	0.903	0.03	Age
Thalamus	92	0.28 (−0.51 to 1.06)	0.39	84	0.71	0.482	0.15	Age, ICV

Results from linear mixed-effects models in individuals with chronic stroke of sensorimotor impairment (top) compared to activity limitations (bottom). Results in bold indicate significance with a Bonferroni correction for multiple comparisons (*P* < 0.004). The beta coefficient for sensorimotor impairment/activity limitations (beta) with 95% confidence interval (CI), along with the sample size (*n*), standard error (SE), degrees of freedom (df), standardized effect size (*d*), *t*-value, and uncorrected *P*-value are reported, in addition to significant fixed covariates, including age, sex and intracranial volume (ICV).

In chronic stroke, we further analysed the differences between individuals with left hemisphere stroke (LHS, *n* = 214) versus right hemisphere stroke (RHS, *n* = 190) by including lesioned hemisphere as an interaction term in the model. There were no significant effects of the side of the lesioned hemisphere on the relationship between sensorimotor behaviour and subcortical volumes, and no main effects of the lesioned hemisphere (see Supplementary material). Inclusion of the lesioned hemisphere into the model did not change the main effects of sensorimotor behaviour. We also examined whether there were differences in behavioural scores for LHS and RHS groups. The median sensorimotor behaviour score in LHS was 0.80 (IQR = 0.39) and in RHS was 0.74 (IQR = 0.49). A Wilcoxon test showed no significant effect of lesioned hemisphere between groups (*P* = 0.29, effect size *r* = 0.053).

Finally, an exploratory analysis of the entire cohort (*N* = 828) demonstrated significant relationships between worse sensorimotor behaviour and smaller volumes of the ipsilesional thalamus (*d *=* *0.33, *P* = 0.001), putamen (*d *=* *0.33, *P* < 0.001), and nucleus accumbens (*d *=* *0.23, *P* = 0.004), and a larger lateral ventricle volume (*d* = −0.23, *P* = 0.001; see Supplementary materials).

## Discussion

We report the first international, multi-site pooled analysis with individual patient data using high-resolution structural brain imaging in stroke rehabilitation research and the largest study to date relating spared subcortical brain volumes to post-stroke sensorimotor behaviour. We identified novel, significant relationships between worse post-stroke sensorimotor behaviour and smaller volumes of spared deep grey matter structures, including the ipsilesional thalamus, putamen, and nucleus accumbens, as well as general atrophy as indexed by enlargement of the ipsilesional lateral ventricle. Notably, analyses included only non-lesioned structures, and significant relationships were found only in the ipsilesional hemisphere. These findings suggest that, post-stroke, secondary subcortical brain alterations related to sensorimotor behaviour occur most prominently in the hemisphere directly affected by the stroke. This was observed despite the fact that, after stroke, atrophy and reorganization has been observed bilaterally.^39^ The identification of sensorimotor relationships with these specific ipsilesional subcortical nuclei may provide novel targets to improve stroke outcomes.

Our results support the hypothesis that different non-lesioned deep grey structures serve distinct roles in subacute versus chronic stroke, which is not surprising given the cascade of neurobiological and neuroinflammatory processes that occur early after stroke.^40^^,^^41^ Within 90 days after stroke, only the ipsilesional thalamus showed detectable associations with post-stroke sensorimotor behaviour, in line with recent research showing marked thalamic atrophy, especially within the first three months post-stroke.^39^ A smaller thalamic volume could reflect cell loss and thalamic dysfunction, thereby limiting resources crucial for early recovery.^4^^,^^39^ Importantly, we found that this relationship is not only present but stronger in the first 21 days post-stroke. As non-lesioned brain volumes within 6 weeks after stroke are assumed to be similar to those before the stroke,^21^ this finding suggests that larger thalamic volumes prior to stroke could provide a neuroprotective effect. Thalamic atrophy was recently associated with loss of extrinsic and intrinsic connectivity between the thalamus and the rest of the brain, suggesting that thalamic measures may serve as an index of global brain function.^42^ Future research using longitudinal datasets with greater spatial specificity could relate changes in specific thalamic nuclei to sensorimotor recovery to identify targets for neuroprotective or early stroke therapies.

Although further research is needed to pinpoint which thalamic nuclei are specifically involved with sensorimotor deficits reported here, we hypothesize that nuclei involved in motor (e.g. ventral anterior nucleus, ventrolateral nucleus), sensory (e.g. ventral posteromedial nucleus, ventral posterolateral nucleus), as well as higher order thalamic regions such as the lateral posterior nucleus, which is involved in integrating sensory input with cognitive functions, should be related. Finally, a critical line of future research is the evaluation of isolated thalamic infarctions due to small arterial vessel disease and the possible relationship between these infarctions and post-stroke sensorimotor behaviour.^43^

In chronic stroke, reduced volumes of the ipsilesional putamen and nucleus accumbens were consistently associated with worse sensorimotor behaviour. General atrophy, as indexed by a larger ipsilesional ventricle volume, was also negatively associated with sensorimotor behavioural measures. This is the first large-scale validation showing volume of these specific structures as correlates of sensorimotor behavioural outcomes in chronic stroke. This finding augments existing stroke literature, which has typically examined direct damage to combine subcortical regions, without differentiating roles of the individual basal ganglia nuclei and thalamus. Here, we specifically identify the putamen and nucleus accumbens, which are key components of corticostriatal and mesolimbic circuits, and which both represent key dopaminergic targets in the brain.

Specifically, within the corticostriatal circuit, the putamen receives direct cortical signals from the primary motor, premotor, and sensory cortices and relays them to the thalamus to modulate motor control. Interestingly, although the caudate also relays input to the thalamus, it receives its inputs from multimodal association cortices and visual regions—not primary motor regions—and did not have a significant brain–behaviour relationship in our analyses. This distinction suggests that post-stroke sensorimotor behaviour is primarily associated with subcortical nuclei specifically receiving direct sensorimotor input. In line with this, we found that smaller putamen volumes related to both worse sensorimotor behaviour generally and impairment specifically, as evidenced by the association with the FMA-UE in chronic stroke. This finding is in line with previous work showing that direct damage to the putamen relates to post-stroke gait impairment,^44^ upper limb impairment^45^ and spasticity,^46^ all deficits which overlap with the behavioural measures used here. In addition, secondary atrophy of the putamen has been reported after cortical stroke and is associated with infarct volume^47^ and post-stroke cognitive deficits.^48^ The relationship between chronic sensorimotor behavioural deficits and atrophy of the ipsilesional putamen after stroke, however, has not previously been reported. As atrophy of the putamen has been associated with a wide variety of neuropsychiatric and neurodegenerative disorders,^49^ including Alzheimer’s disease,^50^ multiple sclerosis, attention deficit disorder^12^ and Huntington’s disease,^10^ it is possible that the integrity of the putamen is required not only for specifically sensorimotor behaviour but also, more generally, for overall healthy brain functioning.

While the ipsilesional nucleus accumbens was significantly related to chronic sensorimotor behaviour in general, it was neither related to sensorimotor impairment (FMA-UE) nor to activity limitation. However, the analyses on impairment and activity limitations had less statistical power to detect relationships. The nucleus accumbens is a key component of the ventral striatum and implicated in fear, stress, and anxiety disorders^51^ as well as the dopaminergic modulation of reward-based behaviours.^52^ As such, this region may impact more complex aspects of motor performance, such as motivation, engagement, and participation, which may not be reflected in metrics of impairment or activity. A number of studies show decreases in ventral striatal processes such as reward sensitivity, motivation, and apathy after stroke,^53^ and post-stroke hypoactivity in the nucleus accumbens has been identified during reward-based decision-making tasks.^54^ Thus the nucleus accumbens may affect sensorimotor behaviour by influencing reward and motivation,^55^ which could impact use of the affected limb in daily tasks. Pharmacological methods to modulate the dopaminergic system and promote motor recovery following stroke have been widely studied, with dopamine expected to influence multiple domains of behaviour, including motor control, motor learning and affective disorders. However, individual outcomes from pharmacological methods vary widely.^56^ Future research may investigate whether individual differences in the volume and connectivity of the nucleus accumbens predict who may benefit from dopaminergic treatment.

In chronic stroke, we also detected an association between an enlarged ipsilesional lateral ventricle and poor sensorimotor behaviour. This relationship was only significant at the chronic stage and was exclusive to the ipsilesional lateral ventricle, which may be due to hydrocephalus ex vacuo. Ventricular enlargement post-stroke may also be influenced by small vessel disease (i.e. leukoaraiosis), although this is typically observed bilaterally.^19^ Enlargement of the bilateral lateral ventricles has also been associated with generalized brain atrophy that occurs during ageing and with impaired cognitive function.^57^ The contrast between ipsilesional and contralesional ventricles may provide unique insight into the specific impact of the stroke versus general ageing on chronic stroke sensorimotor outcomes.

Our results also suggest that there are distinct brain–behaviour relationships for different ICF dimensions of sensorimotor behaviour. Chronic motor impairment, as measured by the FMA-UE, was associated with a smaller ipsilesional putamen and larger ipsilesional ventricle, which may provide an indication of corticostriatal circuit integrity as well as more general brain functions essential for sensorimotor control. In contrast, there were no subcortical associations with activity limitations in the current study, possibly related to the smaller sample size. Activity limitations may also be more strongly related to the integrity or function of distributed regions across whole brain networks rather than subcortical structures,^58^^,^^59^ given that functional performance can be influenced by psychosocial factors to a greater degree than impairment measures.

Findings did not indicate a significant effect of lesioned hemisphere on the relationship between chronic sensorimotor behaviour and spared subcortical volumes. These results are surprising, given that the large majority of patients were likely left hemisphere dominant for motor control, and previous research has identified specialized hemispheric in sensorimotor control after stroke.^25^ However, previous research has primarily focused on cortical regions and functional activity, rather than subcortical structures. Side of stroke injury may not directly impact sensorimotor relationships with spared subcortical volumes.

Finally, the current results represent the first large-scale, multi-site analysis utilizing harmonized high-resolution brain imaging and behavioural measures in the field of stroke rehabilitation. The fact that the current results, using diverse stroke rehabilitation data, fit with existing literature and reveal new findings is further confirmation that such an approach is not only feasible and effective, but also beneficial for moving the stroke rehabilitation field forward.

### Limitations and future directions

A key limitation of pooling multi-site data is inconsistent variables across cohorts, limiting subgroup analyses and reducing the number of included covariates. Models only included the covariates age, sex and intracranial volume; however, many additional demographic variables, such as duration and type of rehabilitation received, handedness, race, educational level and comorbidities, may influence these relationships. Although sex was not a significant covariate in the majority of our analyses, it is also worth noting that previous research has shown that women differ from men in the distribution of risk factors and stroke subtype, stroke severity, and outcomes.^60^^,^^61^ Future work with should more carefully examine the role of sex in post-stroke sensorimotor outcomes. In addition, larger sample sizes for different sensorimotor outcome measures would provide greater support for the current findings. Related, small high-resolution MRI samples (*n* < 50) at earlier time points of stroke (i.e. ≤7 days, defined as acute^20^) with sensorimotor behavioural outcomes limited our ability to specifically examine acute brain–behaviour relationships or to examine relationships between impairment versus activity limitations in acute or subacute stroke in the current analysis. The ENIGMA Stroke Recovery Working Group recommends following consensus guidelines for greater harmonization of prospectively collected data to facilitate more precise pooled analyses across all times after stroke.^14^^,^^62^

Lesion overlap with subcortical regions and poor segmentation of subcortical regions due to lesion-induced distortions resulted in a variable sample size for each ROI, potentially limiting the power to detect relationships in regions with smaller samples. Furthermore, exclusion of individuals with lesioned or incorrectly segmented ROIs may have disproportionately excluded individuals with larger lesions, who may be more severely affected. This could have biased the sample towards more mild-to-moderately impaired patients. Future studies using information about the lesions (lesion location, volume, and overlap) derived from accurately segmented lesion masks for each observation could address these issues. In addition to lesion information, additional quantification of neuroimaging markers of intracranial small vessel disease, such as white matter hyperintensities and perivascular spaces, could provide a deeper understanding of the relationship between small vessel disease and the observed results.

Finally, many of these subcortical regions are also critical for and related to post-stroke cognition, mood, sleep, learning and other traits of interest. While this analysis was limited to sensorimotor behavioural measures to maximize available data for analysis, these findings may not be unique to sensorimotor behaviour. Future studies should assess the relationship between these subcortical volumes and additional stroke outcome measures.

## Conclusion

This international collaborative analysis revealed significant relationships between post-stroke sensorimotor behaviour and volumetric measures of the residual ipsilesional thalamus, putamen, nucleus accumbens, and lateral ventricle at different times after stroke—brain metrics that may reflect overall brain health and network integrity and could lead to the identification of novel neural targets for stroke rehabilitation.

## Supplementary material

Supplementary material is available at *Brain Communications* online.

## Supplementary Material

fcab254_Supplementary_Data

## Appendix

**Current ENIGMA Stroke Recovery Working Group Member List (as of 10/05/2021)**

Nerisa Banaj, Giuseppe Barisano, Lee Baugh, Adrià Bermudo Gallaguet, Anup Bhattacharya, Bavrina Bigjahan, Michael Borich, Lara Boyd, Amy Brodtmann, Truman Brown, Cathrin Buetefisch, Winston Byblow, Jessica Cassidy, Charalambos Charalambous, Valentina Ciullo, Alison Cloutier, James Cole, Adriana Conforto, Richard Craddock, Steven Cramer, Rosalia Dacosta Aguayo, Julie DiCarlo, Michael Dimyan, Martin Domin, Miranda Donnellly, Adrienne Dula, Matthew Edwardson, Natalia Egorova, Elsa Ermer, Mark Etherton, Wuwei Feng, Kelene Fercho, Jennifer Ferris, Fatemeh Geranmayeh, Chris Gregory, Shahram Hadidchi, Colleen Hanlon, Leticia Hayes, Kathryn Hayward, Jess Holguin, Brenton Hordacre, Darryl Hwang, Neda Jahanshad, Keith Jamison, Julia Juliano, Steven Kautz, Mohamed Salah Khlif, Bokkyu Kim, Hosung Kim, Amy Kuceyeski, Catherine Lang, Jenny Lee, Sook-Lei Liew, David Lin, Jingchun Liu, Bethany Lo, Keith Lohse, Martin Lotze, Bradley MacIntosh, John Margetis, Daniel Margulies, Maria Mataro, Keith McGregor, Feroze Mohamed, Jan Nordvik, Emily Olafson, Alexandre Perera-LLuna, Matthew Petoe, Aaron Phillips, Fabrizio Piras, Sharmila Raju, Ander Ramos-Murguialday, Kate Revill, Pamela Roberts, Andrew Robertson, Jane Rondina, Natalia Rost, Nerses Sanossian, Heidi Schambra, Christian Schranz, Nicolas Schweighofer, Na Jin Seo, Farshid Sepehrband, Mark Shiroishi, Julia Simon, Surjo Soekadar, Gianfranco Spalletta, Shraddha Srivastava, Jill Stewart, Cathy Stinear, Anisha Suri, Myriam Taga, Wai Kwong Tang, Gregory Thielman, Vincent Thijs, Sophia Thomopoulos, Paul Thompson, Daniela Vecchio, Steven Warach, Nick Ward, Emilio Werden, Lars Westlye, Roland Wiest, Carolee Winstein, George Wittenberg, Steven Wolf, Kristin Wong, Chunshui Yu, Artemis Zavaliangos-Petropulu.

## Abbreviations

ARAT: Action Research Arm Test
CI: confidence interval
df: degrees of freedom
ENIGMA: Enhancing NeuroImaging Genetics through Meta-Analysis
FMA-UE: Fugl-Meyer Motor Assessment of Upper Extremities
ICF: International Classification of Functioning, Disability, and Health
ICV: intracranial volume
IQR: interquartile range
LHS: left hemisphere stroke
RHS: right hemisphere stroke
ROI: region of interest
SE: standard error
T1w: T_1_-weighted
WMFT: Wolf Motor Function Test
